# Tailoring bilberry powder functionality through processing: Effects of drying and fractionation on the stability of total polyphenols and anthocyanins

**DOI:** 10.1002/fsn3.930

**Published:** 2019-02-07

**Authors:** Gabriel Oliveira, Lovisa Eliasson, Maria Ehrnell, Evelina Höglund, Thomas Andlid, Marie Alminger

**Affiliations:** ^1^ Department of Biology and Biological Engineering, Food and Nutrition Science Chalmers University of Technology Gothenburg Sweden; ^2^ RISE Research Institute of Sweden, Agrifood and Bioscience Gothenburg Sweden

**Keywords:** anthocyanins, bilberry, fractionation, powder, press cake

## Abstract

Bilberries are a rich natural source of phenolic compounds, especially anthocyanins. The press cake obtained during the processing of bilberry juice is a potential source of phytochemicals. The objective of this study was to evaluate different drying techniques and the fractionation of bilberry press cake powder toward obtaining phenolic‐rich ingredients for incorporation into value‐added food products. The derived powders were dispersed in water and dairy cream, to investigate the effects of drying and fractionation on the dispersibility and solubility of phenolic compounds. The drying techniques, hot air drying and microwave drying, applied on bilberry press cake reduced the content of total phenolics and anthocyanins. The degradation was, however, consistently small and similar for both techniques. The major anthocyanins detected in the samples were stable during drying and fractionation treatments. Fractionation of the press cake powder affected the total apparent phenolic content and composition of the different fractions. The highest phenolic content (55.33 ± 0.06 mg g^−1^ DW) and highest anthocyanin content (28.15 ± 0.47 mg g^−1^ DW) were found in the fractions with the smallest particle size (<500 μm), with delphinidin‐3‐O‐galactoside being the most abundant anthocyanin. Dispersibility of all dried powder samples was higher in dairy cream than water, and the highest level of anthocyanins was measured in samples from the powder with the smallest particle size (<500 μm), dispersed in cream. The application of drying, milling and fractionation was found to be a promising approach to transform bilberry press cake into stable and deliverable ingredients that can be used for fortification of food products with high levels of phenolic compounds.

## 1 INTRODUCTION

Berry press cake is a natural and abundant by‐product of the processing of berry juices. The berry press cake, which may account for up to 20%–30% of the initial weight of the berries and which includes skins, pulp, seeds, and edible parts of stems, contains vitamins, fiber, polyphenols (including anthocyanins; Khanal, Howard, Brownmiller, & Prior, 2009). Despite its high contents of valuable compounds, press cake is generally considered a waste by‐product and is typically recycled as animal feed, composted or discarded due to challenges associated with short shelf life, texture, and flavor (Rohm et al., 2015). Bilberries (*Vaccinium myrtillus* L.) are a rich natural source of flavonoids, especially anthocyanins, which are concentrated to a greater extent in the fruit peel (20 mg/g) than in the fruit pulp (1 mg/g; Riihinen, Jaakola, Kärenlampi, & Hohtola, 2008). Therefore, bilberry press cakes represent an important and attractive source of phenolic compounds for use in food products.

Due to the high moisture content of press cake (around 70%), initial processing is needed to avoid spoilage and to ensure a satisfactory shelf life. Hot air drying (HAD), also referred to as convective drying, is widely used in the food industry (Askari, Emam‐Djomeh, & Mousavi, 2009). During this process, hot air transfers heat to the wet fruit by convection, and thereafter, the interior part of the fruit is heated by conduction. Simultaneously, moisture migrates to the fruit surface. While this technique is easily accessible, it often requires long drying times and a high air temperature, which makes it energy‐demanding (Fellows, 2000; Karam, Petit, Zimmer, Djantou, & Scher, 2016). However, since the processing conditions during drying greatly influence the quality attributes of the product, including its nutritional and sensorial characteristics, it is important to adapt the processing conditions to minimize any adverse effects (Zielinska, Sadowski, & Błaszczak, 2016). Microwave drying is an interesting alternative, as it results in direct heating of the product without the need to heat the surrounding air. This enables fast heating rates and reduced processing time, compared to conventional techniques, which could provide beneficial effects on quality and economical aspects. Microwave heating results in internal heating of the product, creating a pressure‐driven flow that mobilizes the moisture to the surface of the product, thereby minimizing structural changes in the product (Chou & Chua, 2001; Datta, 2001).

Berries are known to be rich in phenolic compounds, such as anthocyanins, hydroxybenzoic acid, ellagitannins, and flavonols (Määttä, Kamal‐Eldin, & Törrönen, 2003). Interventional and epidemiological studies have shown that intake of anthocyanins and berry fruits improves insulin sensitivity and reduces the risk of type 2 diabetes (Guo, Yang, Tan, Jiang, & Li, 2016; Stull, Cash, Johnson, Champagne, & Cefalu, 2010). In addition, the efficacies of the biological activities seem to be related to structural differences of the anthocyanins. In particular, the primary anthocyanidins, delphinidins and malvidins, have been reported to improve the profiles of metabolic and cardiovascular disease risk biomarkers (Hansen et al., 2005; Hidalgo et al., 2017; Overall et al., 2017). The stability of polyphenols is influenced by, for instance, temperature, pH, light intensity, and oxygen availability (Furtado, Figueiredo, Neves, & Pina, 1993), and during treatments, drying, temperature and time are important factors determining the degradation of anthocyanins. As the temperature rises, the stability of anthocyanins decreases, mainly due molecular ring ruptures or polymerization (Nayak, Berrios, Powers, & Tang, 2011; Weber & Larsen, 2017). However, depending on their structures, anthocyanins can show different temperature sensitivity, for example, Nayak et al. (2011) have shown that cyanidin bonds with different glucoside groups have different half‐life when heated at 95°C.

Although berry press cakes have been frequently referred to as a very valuable fruit processing by‐product, more studies are needed to gain information how fresh berry press cakes can be transformed into a stable and deliverable raw material without losing functionality by degradation of polyphenols because of the heat load during the removal of moisture. Milling of press cakes into powders with different particle sizes can be used to modify the product properties, for example, the dispersibility of the powder in liquid‐based foods (Camire, Dougherty, & Briggs, 2007; Da Costa, Felipe, Maia, Hernandez, & Brasil, 2009; Park, Imm, & Ku, 2001). Therefore, the hypothesis underlying the present study was that tailoring of the properties of bilberry press cake powder by mild drying and fractionation can be used to improve the functionalities of bilberry powder for utilization in food products.

The objective of this study was to evaluate the effects of two drying techniques—hot air drying (HAD) and microwave‐assisted hot air drying (MWD)—on total phenolic content (TPC) of bilberry press cake powder (with focus on anthocyanin composition) and of fractionation into three different particle sizes. The goals were to identify the least destructive method that would preserve berry quality and to study the effects of particle size on the dispersibility of the powders in water and dairy cream.

## 2 MATERIALS AND METHODS

### 2.1 Chemicals

All chemicals and solvents used were of analytical grade. Methanol (LC‐MS grade) was purchased from Fisher Scientific (Loughborough, UK), trifluoroacetic acid and chloroform (HPLC grade) from Sigma‐Aldrich Chemical Co. (St. Louis, MO, USA), gallic acid from Fluka Biochemika (Stockholm, Sweden), hydrochloric acid from Acros Organics (Germany), and formic acid from Scharlau (Barcelona, Spain).

Anthocyanin standards cyanin chloride, malvin chloride, malvidin‐3‐O‐glucoside chloride, peonidin chloride, petunidin‐3‐O‐glucose chloride, cyanidin‐3‐O‐glucoside chloride, malvidin chloride, cyanidin chloride, delphinidin chloride, cyanidin‐3‐O‐arabinoside chloride, delphinidin‐3‐O‐galactoside chloride, and peonidin‐3‐O‐glucoside chloride were all purchased from Extrasynthese (Genay, France).

### 2.2 Bilberry raw material and press cake production

**Figure 1:**
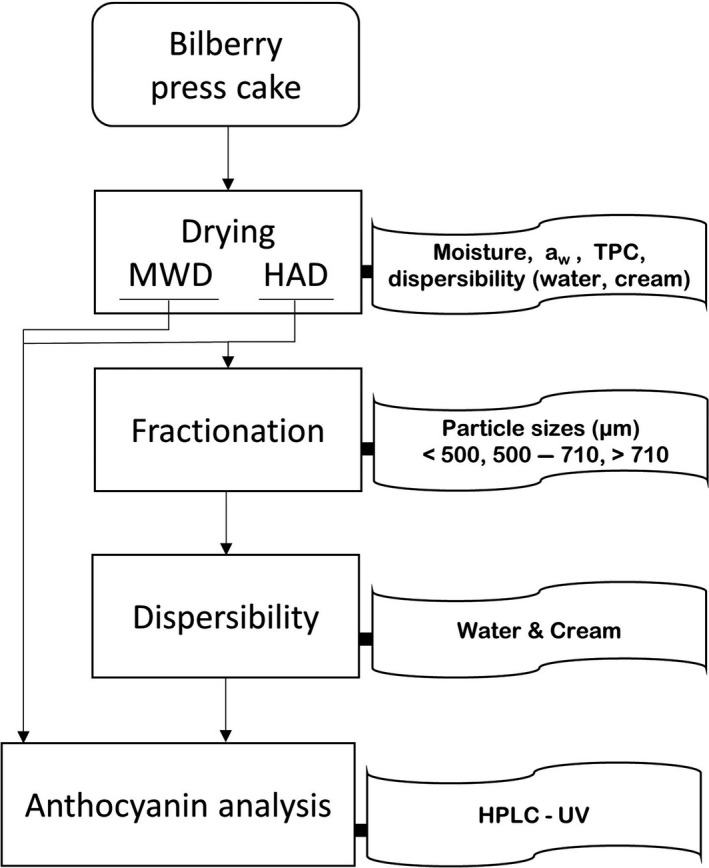
Overview of the material and methods. HAD refers to hot air drying and MWD to microwave drying. TPC refers to total phenolic compounds and aw to water activity

Bilberries (*V. myrtillus* L.) were supplied by Olle Svensson AB (Olofström, Sweden) and stored in the dark at −40°C prior to the experiments. Figure 1 shows a schematic overview of the material and methods used in the study.

The press cake was produced in laboratory scale using a mechanical press (Hafico, Germany), applying a maximum pressure of 200 kg/cm. The bilberries (0.9 kg) were thawed in darkness at 6°C for 16 hr before pressing. The berries were cold‐pressed without enzymatic treatment, resulting in a press cake with a moisture content of 71.2 ± 0.69% and a press cake yield of 18.9 ± 0.94%.

### 2.3 Drying and milling

Hot air drying of the sample was carried out in a conventional hot air oven (Garomat 142; Electrolux AB, Stockholm, Sweden) with an air velocity of 6.1 m/s. Bilberry press cakes were distributed into two trays (21 × 30 cm), each containing 250 g of berry material, and dried at 40°C protected from light until a water activity of about 0.5 was achieved. The drying times required for each sample were determined by assessing (on a continuous basis), the decrease in material weight and the water activity during pretrials. The dried material was stored in sealed polyamide/polyethylene plastic pouches and protected from light at −40°C until milling. MWD was performed according to the procedure described by Kerbstadt, Eliasson, Mustafa, and Ahrné (2015). In brief, the microwave system consisted of a microwave cavity (Tivox AB, Tidaholm, Sweden) and an air‐heating unit (Honeywell INU Control AB, Borås, Sweden). The microwaves were generated by a MagDrive‐1400 (Tivox, Tidaholm, Sweden), and the microwave power was regulated automatically by the software MagDrive ver. 3.1 (Tivox Masik AB, Tidaholm, Sweden), depending on temperature of the press cake material. The mean temperature was recorded using four fiber‐optic temperature probes (Neoptix Inc, Québec, Canada). During the drying process, the microwave output power was regulated in four different steps: (a) maximum 1,000 W for 1 min to quickly reach the treatment temperature, (b) maximum 750 W for 40 min, (c) maximum 400 W for 20 min, and (d) maximum 200 W for the remaining drying time. To keep the desired treatment temperature, the output power was reduced along the drying when the moisture content of the product was reduced.

For both drying techniques, around 500 g of bilberry press cake was dried at 40°C in the dark, until a water activity of about 0.5 was achieved, at which point a constant weight had been obtained.

The dried bilberry press cake (75 g) was milled in a knife mill (Grindomix GM200; Retsch GmbH, Haan, Germany) at 800 *g* for 10 s. After milling, the sample was stored in sealed polyamide/polyethylene plastic pouches in the dark at −80°C until analysis.

### 2.4 Moisture content and water activity

The moisture content was determined in a vacuum oven (Sanyo Gallenkamp, Loughborough, UK) using a gravimetrical method. The sample (2 g) was dried at 70°C and 900 mbar, until constant weight. The water activity was analyzed in triplicate using the Aqua Lab 4TE instrument (Decagon Devices, Pullman, WA, USA).

### 2.5 Particle size distribution and fractionation

A vibratory sieve shaker (Analysette 3; FRITSCH GmbH, Ida‐Oberstein, Germany) with mesh sizes of 125, 250, 500, 710, and 1250 μm was used to determine the particle size distribution of the HAD and MWD press cake powders. To separate the HAD powder into three different fractions, sieves of mesh sizes 500 and 710 μm were used. The powder (20 g) was sieved for 10 min, at an amplitude of 1.5 mm and an interval of 10 s.

Visualization of the three particle size fractions was done with a camera equipped with a macrolens (EF‐S 60 mm and EOS 600D; Canon, Tokyo, Japan).

### 2.6 Dispersibility

Dispersibility of powders in food matrices is an important feature of powder ingredients because recovering the unique particle properties as nutritional quality, flavor, taste, and color can add value to other food products. Dispersibility of powders is also important to predict their behaviors for specific end usage.

In this study, dispersibility was defined as the weight of powder that remained suspended in the liquid phase after mixing it with a liquid and allowing a subsequent resting time.

The dispersibility of the bilberry powders was studied in water and dairy cream (15% fat, purchased in a local supermarket) using a method adapted from Shittu and Lawal (2007) and Jaya and Das (2004). Briefly, 15 g of hot air‐dried powder was added to 300 g of liquid (water or cream) and stirred for 5 min using a magnetic stirrer (RCT basic; IKA GmbH, Staufen, Germany) and a magnet covering the bottom of a 600‐ml glass beaker. After stirring, the suspension was left to stand for 12.5 min to allow the suspended particles to settle, after which 7 g of the supernatant was carefully pipetted into a Petri dish. The Petri dishes with supernatants were placed in a vacuum oven (Fistreem International Ltd., Loughborough, United Kingdom) at 80°C and 900 mbar and dried until constant weight. Samples (duplicate) of dairy cream without powder addition were dried in the same way, to permit subtraction of the weight of the cream components, thus allowing the determination of the percentage of dispersed particles (w/w). Samples from the supernatants were removed by transferring approximately 30 g into Falcon tubes and stored at −80°C until analysis of TPC.

### 2.7 Total phenolic content

The TPC was determined by the Folin–Ciocalteu method based on the publications of Howard, Clark, and Brownmiller (2003) and Barnes, Nguyen, Shen, and Schug (2009), with some modifications. Briefly, freeze‐dried berry powder and samples from the dispersibility experiments (0.2 ± 0.015 g) were extracted in triplicate with acidified methanol (MeOH:H_2_O [70:30 mix] plus 1% trifluoroacetic acid). After the addition of Folin–Ciocalteu reagent, the extracts were analyzed spectrophotometrically against a standard curve of gallic acid, measuring the absorbance at 765 nm in a Safire 2 plate reader (TECAN) with the Magellan software. The TPC is presented as gallic acid equivalent (GAE) per g of dry weight (mg GAE g^−1^ DW).

### 2.8 Extraction of anthocyanins

Freeze‐dried bilberry powder and samples from the dispersibility experiment were extracted using acidified methanol according to Bunea et al. (2013) and You et al. (2011) methods, with some adaptations. Freeze‐dried samples (0.200 ± 0.015 g) were mixed by vortexing for 30 s with 3 ml of methanol that contained 0.3% HCl (v/v) in a glass tube with a screw cap. Nitrogen gas was used to flush the air from the tube. The extraction mixture was placed in the dark at 4°C for 18 hr. After sonication for 15 min at 20°C, 37 kHz (S15 Elma Sonicator, Elma Schmidbauer GmbH), the samples were centrifuged at 2,000 *g* for 10 min and the supernatant fluid was collected. Re‐extraction was carried out by adding 3 ml of acidified methanol to the pellet, followed by vortexing, centrifugation, and finally pooling the supernatants from each extraction. The supernatants (6 ml) were centrifuged at 4,000 *g* for 15 min and stored at −20°C until analysis.

For the extraction of anthocyanins from bilberry powder samples dispersed in cream, an adaptation of the method described by Jing and Giusti (2005) was used. The initial extraction steps were similar to those in the method described above but with the modification that 4 ml of acidified methanol was used and after shaking for 15 s, the mixture was stored in the dark at 4°C for 18 hr. After sonication for 15 min and centrifugation, 3 ml of chloroform was mixed thoroughly with the supernatant in a test tube with a screw cap. After the addition of 3 ml of ultrapure water and mixing, the mixture was centrifuged at 4,500 *g* and 4°C. The upper aqueous phase containing the anthocyanins was transferred to a new tube, and the extraction was repeated again with the remaining chloroform phase by adding 3 ml of ultrapure water, followed by centrifugation. The obtained aqueous phases were combined.

### 2.9 HPLC‐UV analysis of anthocyanins

The high‐performance liquid chromatography (HPLC) system consisted of a quaternary gradient pump (Jasco PU‐2089 Plus; Jasco Inc., Easton, MD, USA), a cooled (8°C) autosampler (Jasco AS‐2057 Plus), and a UV detector operating at 520 nm (Shimadzu SPD‐10A UV‐Vis detector; Shimadzu Corp., Kyoto, Japan). A Jasco ChromNAv software was used to control the HPLC system and for data processing. Separation of the individual anthocyanins was achieved with a Phenomenex Luna C_18_ column (150 × 3.0 mm, 3‐μm) at 40°C, with 5% aqueous formic acid (A) and methanol (B) as eluents. A linear gradient was applied at a flow rate of 0.5 ml/min with an increase from 13% to 25% of eluent B over 25 min, followed by an increase to 50% of eluent B over 2 min, and isocratic elution at 50% with eluent B for 5 min. The column was reconditioned (13% eluent B) for 5 min prior to injection of the next sample. The injection volume was 10 μl. Identification was based on retention time, UV spectra, comparison with commercial standards, and the relative retention values from the literature (Buchert et al., 2005; Kähkönen, Heinämäki, Ollilainen, & Heinonen, 2003; Lätti, Riihinen, & Kainulainen, 2008). For quantification of the identified compounds, a five‐point external standard calibration curve was prepared by dissolving the anthocyanin standards in aqueous methanol (50% v/v). The linearity of the standard curve was found to be acceptable (*R* ^2^ > 0.998). Internal standards were prepared by spiking some extracted samples with cyanin chloride, to evaluate any fluctuation in the response, and malvidin‐3‐O‐glucoside chloride was used to evaluate matrix effects.

### 2.10 Statistical analysis

One‐way analysis of variance (ANOVA) and *Tukey's* honestly significant difference (HSD) test were applied to compare the samples. Statistical significance was defined as *p* < 0.05. Statistical analyses were performed using the IBM^®^ SPSS^®^ Statistics ver. 24 software.

## 3 RESULTS AND DISCUSSION

The press cake was dried to constant weight, corresponding to a water activity of about 0.5, and a moisture content of 8% (Table 1). The drying time was slightly reduced (7%) by applying MWD instead of HAD (7 vs. 7.5 hr). It was expected that the drying with MWD would be more efficient than HAD and the difference in drying time longer. In a previous study, applying MWD and HAD to obtain bilberry press cake with a moisture content of 17%, MWD was reported to reduce the drying time by 40%, compared with HAD (Höglund et al., 2018). A possible explanation for the different results can be that the powders in the present study were dried to a lower moisture content (about 8%), and since microwaves primarily affect the water molecules in the samples, the heating caused by microwaves in the MWD process became less prominent as the moisture content decreased.

**Table 1:** Moisture content, drying time to reach constant weight, and water activity of the bilberry powders obtained by different drying techniques and after fractionation

Sample	Moisture (%, w/w)	Drying time (hr)	Water activity
Press cake before drying	71.2 ± 0.69	–	–
MWD, whole powder	8.4 ± 0.14	7	0.52 ± 0.006
HAD, whole powder	8.4 ± 0.18	7.5	0.52 ± 0.003
HAD < 500 μm	8.0 ± 0.19	7.5	0.47 ± 0.001
HAD 500–710 μm	7.3 ± 0.18	7.5	0.49 ± 0.004
HAD > 710 μm	8.1 ± 0.27	7.5	0.51 ± 0.003

*Note*. MWD, microwave‐assisted hot air drying; HAD, hot air drying.

**Figure 2:**
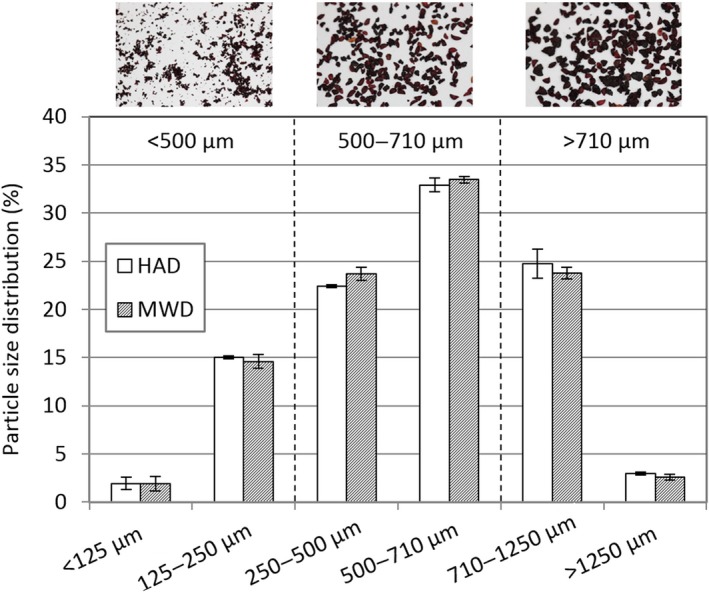
Particle size distribution (%, w/w) of bilberry powders from press cake dried by hot air drying (HAD) and microwave‐assisted hot air drying (MWD). The dotted lines indicate the limits used for fractionation of the powder into three different fractions

A milling time of 10 s was applied to obtain an appropriate size distribution of the particles (based on our previous studies), enabling separation into the three fractions of finely milled powder (<500 μm); seed fraction (500–710 μm); and peel fraction (>710 μm). No difference in particle size distribution was observed between the HAD and the MWD dried press cake samples (Figure 2).

Due to the similar levels of anthocyanins found in the HAD and MWD dried powders (Figure 5) and since that HAD technology is more likely to be suitable for commercial upscaling purposes, a decision was taken to continue the work with only the HAD powder.

The HAD powder was fractionated into three particles sizes with the following distribution: <500 μm (39%); 500–710 μm (33%); and >710 μm (28%; Figure 2).

### 3.1 Dispersibility characteristics of bilberry powders in water and dairy cream

**Figure 3:**
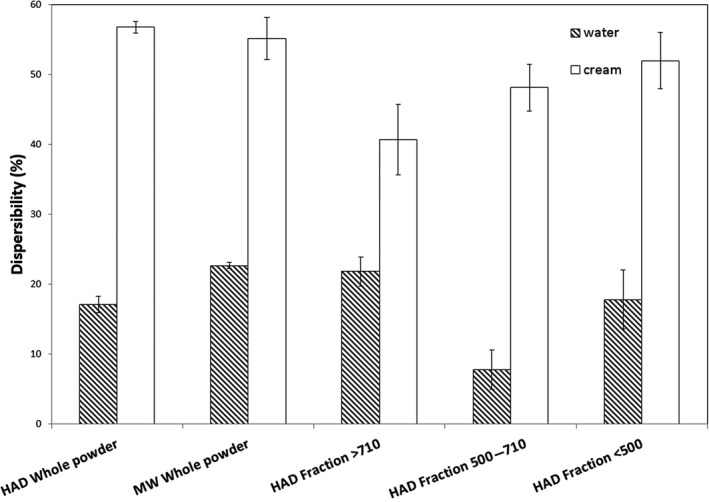
Dispersibility (%, w/w) of powders from microwave‐assisted hot air‐dried (MWD), hot air‐dried (HAD), and fractionated HAD powders from bilberry press cake

As shown in Figure 3, the dispersibility profiles of the MWD and HAD dried whole bilberry powders showed similar behaviors. Separation of the HAD into fractions with different particle size affected the dispersibility characteristics of the fractionated powders.

The dispersibility of the intermediate fraction (500–710 μm) in water was lower than the larger and the smaller fractions, probably due to the high content of seeds in this fraction, which sank to the bottom and was not well dispersed. In cream, however, the structure of the cream matrix supported the dispersion of the seeds. Similar behaviors were noted for the large and small fractions when comparing cream and water. In cream, a trend of higher dispersibility with decreasing particle size was observed, although differences were not significant.

In general, the dispersibility values of the different powders in cream were higher than those in water likely due to that the higher viscosity of the cream matrix better supports the dispersed particles.

### 3.2 Effect of drying treatments on total phenolic and anthocyanin content

The drying techniques applied on bilberry press cake reduced the content of total phenolics and anthocyanins. The degradation was, however, consistently small ranging from 7% (total phenolics) to 19% (anthocyanins).

### 3.3 Total phenolic content in fractionated powders

The total apparent phenolic contents of the dried and fractionated bilberry powders are shown in Table 2. The TPC values varied, with the highest level found for the fraction with the smallest particles (<500 μm) and the lowest for the powder with intermediate particle sizes (500–710 μm). Although the fraction with the largest particles (>710 μm) also contained peel flakes, the TPC was similar to that of the whole dried bilberry powder. This may be explained by a reduced extraction efficiency due to the larger particle size. The presence of particles from the peel, in combination with the small particle size (contributing to enhancing the apparent phenolic content), explains why the highest TPC was noted for the fraction with the smallest particle size. The low TPC in the intermediate size fraction (500–710 μm) is probably linked to the high proportion of intact seeds in this fraction (Aura et al., 2015).

**Table 2:** Total apparent phenolic content of whole berries, press cake, and powders from MWD, HAD, and fractionated HAD powders. Values followed by the same letter were not significantly different (*p* < 0.05) based on Tukey's test

Sample	Total phenolic content (mg GAE g^−1^ DW)
Whole berries before drying	36.54^d^ ± 3.12
Press cake before drying	51.87^ab^ ± 0.38
MWD, whole powder	48.28^bc^ ± 0.61
HAD, whole powder	45.52^c^ ± 1.16
HAD, >710 μm	44.72^c^ ± 1.10
HAD, 500–710 μm	33.62^d^ ± 0.76
HAD, <500 μm	55.33^a^ ± 0.06

*Note*. DW, dry weight; GAE, gallic acid equivalent; HAD, hot air drying; MWD, microwave‐assisted hot air drying.

### 3.4 TPC of bilberry powders dispersed and dissolved in water and cream

Although the lowest TPC was obtained for the intermediate size fraction (500–710 μm; Table 2), the phenolics dispersed well in water, given that there was no significant difference between the levels of phenolics in the whole bilberry press cake and the different fractions dispersed in water (Table 3). This is likely connected to that the intermediate fraction also had the lowest dispersibility (Figure 3), as the intact seeds present in this fraction sedimented. Consequently, the fraction of the powder that was dispersed and dissolved in water was probably enriched in phenolics. This is in agreement with the results obtained for the dispersibility in cream, with the lowest TPC being seen for the intermediate fraction (Table 3). In contrast to the values obtained for water dispersibility, the dispersibility of the intermediate powder fraction was high in cream (Figure 3), which might be due to that the cream matrix prevented the seeds from sinking to the bottom. Since anthocyanins are concentrated in the peel of bilberries (Burdulis et al., 2009; Riihinen et al., 2008), the presence of more seeds did not enhance the apparent total phenolic for this sample. The higher TPC detected in the sample dispersed in water (Table 3), despite its lower dispersibility compared with that in the cream, might be explained by the presence of water‐soluble compounds in the press cake.

**Table 3:** Total apparent phenolic content of powders from MWD, HAD, and fractionated HAD powders from bilberry press cake dispersed in water and cream expressed as mg/g dry weight. Values followed by the same capital letter for water and small letter for cream were not significantly different (*p* < 0.05) based on Tukey's test

Sample	Total phenolic content (mg GAE g^−1^ DW)	
	Water	Cream
MWD, whole powder	26.49^A^ ± 0.92	19.36^ab^ ± 2.25
HAD, whole powder	23.06^A^ ± 0.31	17.43^ab^ ± 3.25
HAD, >710 μm	26.62^A^ ± 2.39	7.26^bc^ ± 1.33
HAD, 500–710 μm	25.55^A^ ± 0.27	4.62^c^ ± 0.27
HAD, <500 μm	26.80^A^ ± 1.69	25.48^a^ ± 5.12

*Note*. DW, dry weight; GAE, gallic acid equivalent; HAD, hot air drying; MWD, microwave‐assisted hot air drying.

In the cream, the TPC of the MWD powder was slightly higher than that of the HAD powder (Table 3). This could be due to particles of the microwave‐dried powder having different structures than HAD particles, which may have promoted the release of polyphenols in the cream. The highest TPC in cream was observed in the fraction with the smallest particle size (<500 μm), while the two fractions with larger particle sizes had significantly lower contents of phenolic compounds. The lower TPCs of the intermediate size fractions (500–710 μm) are likely related to their higher proportions of intact seeds, which contain lower levels of phenolics than the other parts of the press cake (Table 2). The fraction with particles of the smallest size (<500 μm) dispersed in cream had a higher TPC than the whole powder and the larger‐sized particle fractions, probably due to more efficient extraction of phenolic compounds in the cream owing to the larger surface area and ruptured cell walls. In addition, this fraction might also contain broken seeds and might release polyphenols in the forms of ellagitannins and flavonols (Grzelak‐Błaszczyk, Karlińska, Grzęda, Rój, & Kołodziejczyk, 2017) into the cream.

### 3.5 Anthocyanin composition

**Figure 4:**
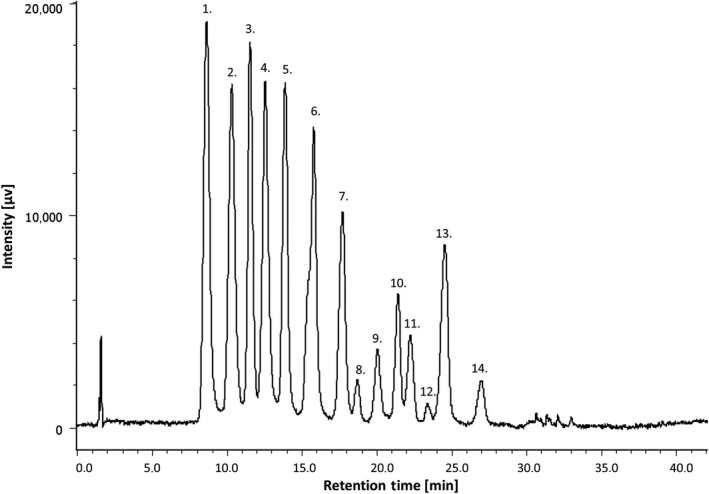
HPLC chromatogram of anthocyanins in freeze‐dried bilberry press cake powder. Peak identification: 1. delphinidin‐3‐O‐galactoside, 2. delphinidin‐3‐O‐glucoside, 3. cyanidin‐3‐O‐galactoside, 4. delphinidin‐3‐O‐arabinoside, 5. cyanidin‐3‐O‐glucoside, 6. cyanidin‐3‐O‐arabinoside, 7. petunidin‐3‐O‐glucoside, 8. peonidin‐3‐O‐galactoside, 9. petunidin‐3‐O‐arabinoside, 10. peonidin‐3‐O‐glucoside, 11. malvidin‐3‐O‐galactoside, 12. peonidin‐3‐O‐arabinoside, 13. malvidin‐3‐O‐glucoside, 14. malvidin‐3‐O‐arabinoside

In the chromatogram obtained from the anthocyanin analyses (HPLC and UV detection) of freeze‐dried bilberry press cake powder, a total of 14 peaks were identified (Figure 4), using standard compounds and data from the literature (Chandra, Rana, & Li, 2001; Jin et al., 2016; Skrede, Wrolstad, & Durst, 2000). The quantification of malvidin‐3‐O‐glucoside, delphinidin‐3‐O‐galactoside, cyanidin‐3‐O‐arabinoside, cyanidin‐3‐O‐glucoside, and petunidin‐3‐O‐glucoside was achieved using calibration curves prepared with external standards. The validation of the method by the spiking of some samples with internal standards showed satisfactory performance, with no response fluctuations or matrix effect, as compared with the external calibration curve.

**Figure 5:**
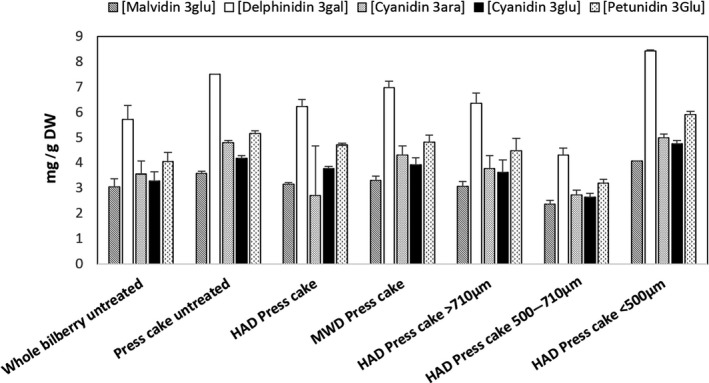
Concentration (mg g^−1^ dry weight) of anthocyanins in bilberry press cake powders after hot air drying (HAD) and microwave drying (MWD) treatments and fractionation of HAD powder (mean ± *SD*,* n* = 3)

**Figure 6:**
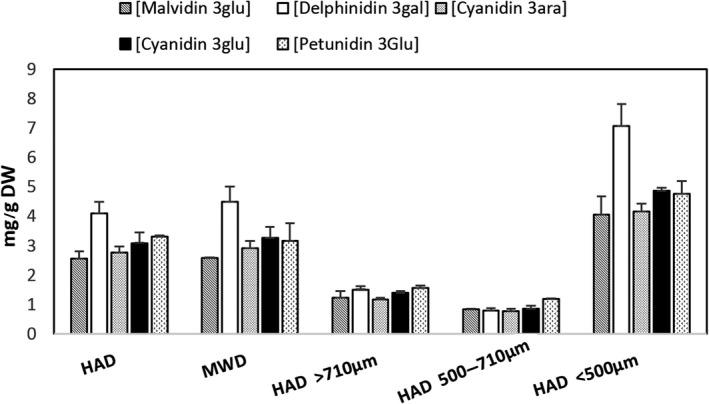
Concentration (mg g^−1^ dry weight) of anthocyanins in bilberry press cake powders after hot air drying (HAD) and microwave drying (MWD) treatments and fractionation of HAD powder dispersed in dairy cream (mean ± *SD*,* n* = 3)

In Figure 5, the total contents of the five most abundant anthocyanins in the HAD, MWD, and fractionated powders are shown. All the bilberry powders showed a similar anthocyanin profile. The most abundant anthocyanin was delphinidin‐3‐O‐galactoside (7.50 ± 0.18 mg g^−1^ DW for untreated press cake), which is in agreement with previous studies reporting that delphinidin glycosides are among the most common anthocyanins in bilberries (Zoratti, Jaakola, Häggman, & Giongo, 2015). The different drying methods (MWD and HAD) did not induce any significant differences in terms of the total anthocyanins or the anthocyanin profile, and as with the TPC, the highest levels of anthocyanins were found in the press cake fraction with the smallest particle size. After dispersion of the different powder fractions in cream, markedly higher levels of anthocyanins and especially the relative amount of delphinidin‐3‐O‐galactoside were quantified in samples from the small particle size fractions (<500 μm) compared to the two fractions with larger particle size (Figure 6).

The total amount of the five anthocyanins in the untreated bilberry press cake powder was 26.3 mg/g (Figure 5). The effect of the fractionation and dispersion on the anthocyanin profile was studied by comparing the total amount of the five identified anthocyanins in the different fractions. The ratio of the sum of all the quantified anthocyanins found in the HAD press cake fraction with smallest particle size to the intermediate fraction was 1.8 (Figure 5).

After dispersion of these samples in cream, the corresponding ratio was 4 (Figure 6). It is possible that the combination of small particle size and the cream matrix promoted interactions between the anthocyanins and other compounds, such as proteins (Cortez, Luna‐Vital, Margulis, & Gonzalez de Mejia, 2017), thereby enhancing the release of anthocyanins in the system. The concentrations of the five quantified anthocyanins (delphinidin‐3‐O‐galactoside, malvidin‐3‐O‐glucoside, cyanidin‐3‐O‐arabinoside, petunidin‐3‐O‐glucoside, and cyanidin‐3‐O‐glucoside) were similar in the intermediate particle fractions with dispersed in cream (Figure 6). The results from analysis of the powder before dispersion were different (Figure 5), possibly due to that the amounts dispersed in cream were low making it difficult to detect differences.

Before dispersion in cream, the anthocyanin content of the large particle size fraction (>710 μm) was similar to that of the whole powder. However, after dispersion, both the content and profile changed, as compared to the whole powder. This probably reflects the lower dispersibility, as well as the large size of the flakes limiting the release of anthocyanins into the solution.

## 4 CONCLUSIONS

The drying techniques, HAD and MWD, applied on bilberry press cake reduced the content of total phenolics and anthocyanins. The degradation was, however, consistently small and similar for both techniques. Milling of bilberry press cake into powders with small particle size (<500 μm) provided the highest apparent content of phenolic compounds. Dispersibility of all dried powder samples was higher in dairy cream than water, and the highest level of anthocyanins was measured in samples from the powder with the smallest particle size (<500 μm), dispersed in cream. The application of drying, milling, and fractionation was found to be a promising approach to transform bilberry press cake into stable and deliverable ingredients that can be used for fortification of food products with high levels of phenolic compounds. In addition, by increasing the specific surface, milling may allow beneficial interactions with a cream matrix. The anthocyanins delphinidin‐3‐O‐galactoside, cyanidin‐3‐O‐arabinoside, cyanidin‐3‐O‐glucoside, malvidin‐3‐O‐glucoside, and petunidin‐3‐O‐glucoside, which together account for the major part of the total phenolics present in the bilberry press cake, were found to be stable during HAD or MWD and fractionation treatments.

## CONFLICT OF INTEREST

The authors declare no conflict of interest.

## ETHICAL STATEMENTS

This study does not involve any human or animal testing.
